# The mitochondrial genome of the deep-sea axiid shrimp, *Eiconaxius baja* (Decapoda: Axiidae)

**DOI:** 10.1080/23802359.2022.2107445

**Published:** 2022-08-10

**Authors:** Ian V. Hughes, Avery S. Hiley, Greg W. Rouse

**Affiliations:** Scripps Institution of Oceanography, University of California San Diego, La Jolla, CA, USA

**Keywords:** *Eiconaxius*, mitogenome, Axiidae, phylogeny

## Abstract

Here, we present the first mitochondrial genome of *Eiconaxius baja*. The mitogenome contains 13 protein-coding genes (PCGs), two rRNA genes, and 22 tRNA genes. The total length of the complete *E. baja* mitochondrial genome is 16,212 base pairs, and the GC content is 26.82%. The gene order is consistent with that of *Eiconaxius serratus*, and most other members of Axiidea. Phylogenetic analysis based on 13 PCGs places *E. baja* sister to *E. serratus* within Axiidae.

The axiid shrimp genus *Eiconaxius* Bate, 1888 comprises more than 30 species found in deep oceans worldwide, often in association with hexactinellid sponges (Komai and Tsuchida 2012; Poore 2018; WoRMS 2022). The classification of *Eiconaxius* has been debated, including being placed in its own family Eiconaxiidae Sakai and Ohta 2005. However, molecular phylogenies and morphological evidence provide support for placing *Eiconaxius* in Axiidae Huxley, 1879 (Tsang et al. 2008; Kou et al. 2020). Although Axiidae was traditionally placed within Thalassinidea Latreille, 1831, molecular phylogenies have challenged the validity of this taxon, and instead position Axiidae within Axiidea de Saint Laurent, 1979 (Tsang et al. 2008; Lin et al. 2012; Tan et al. 2017; WoRMS 2022). Of the 371 accepted species in Axiidea (WoRMS 2022), 11 have published mitochondrial genomes available in GenBank to date. Further molecular data will be useful for confirming the taxonomic placement of *Eiconaxius* and for resolving the relationships among species in the genus.

*Eiconaxius baja* Kensley, 1996 is an eastern Pacific species distributed from northern Baja California to the Channel Islands off California. To date, no genetic data have been available for *E. baja*, and its position within *Eiconaxius* remains unknown. A pair of *E. baja* individuals, resident in a glass sponge (Farreidae Gray, 1872), were collected at 500–1000 m depth on the slope of the San Juan Seamount (33.0391° N 121.0052° W) in October 2020. No ethical approval was required for collection (IACUC, UC San Diego, La Jolla, CA). The specimens were preserved in 95% ethanol and deposited in the Scripps Institution of Oceanography Benthic Invertebrate Collection (https://sioapps.ucsd.edu/collections/bi/, contact: Greg Rouse, grouse@ucsd.edu), under the voucher SIO-BIC C14467. The objects of this study are to provide the first published genetic data for *Eiconaxius baja* and to further help resolve phylogenetic relationships within Axiidea.

DNA was extracted from tissue of the female specimen using the Zymo Research DNA-Tissue Miniprep kit (Zymo Research, Irvine, CA), following the manufacturer’s protocol. Extracted DNA was sequenced on the Illumina Novaseq6000 150 base pairs (bp) platform (Illumina, San Diego, CA) following library preparation by Novogene (en.novogene.com/), generating 7,993,403 paired-end raw reads of 150 bp each.

**Figure 1.**
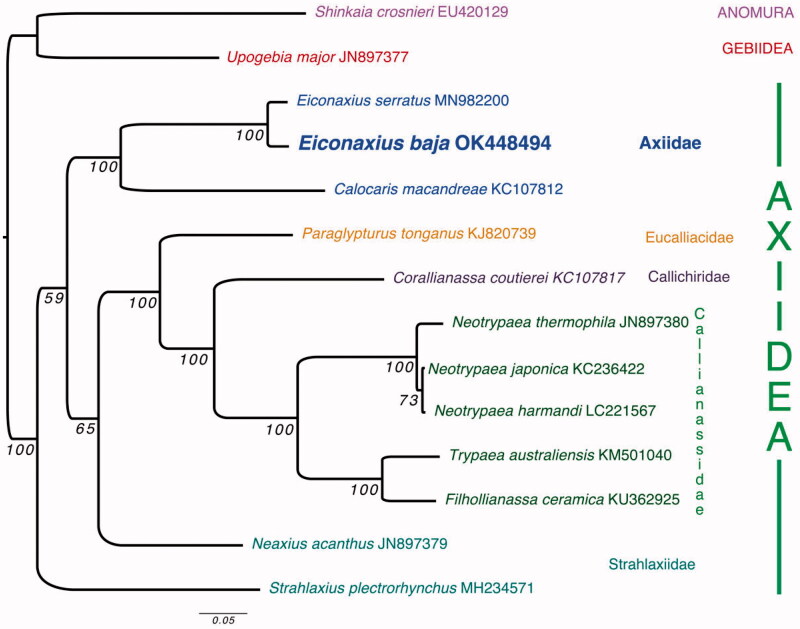
Maximum-likelihood tree based on 13 protein-coding genes for *Eiconaxius baja* and 11 additional members of Axiidea. *Shinkaia crosnieri* and *Upogebia major* were used as outgroups based on Kou et al. (2020). Node labels correspond to ML bootstrap values.

Sequence reads were trimmed (leading and trailing low quality or N bases below quality 3 were removed; reads were scanned with a four-base wide sliding window and deleted when the average quality per base dropped below 15; and reads under 36 bp long were dropped) and cleaned of adapters using Trimmomatic v. 0.39 (Bolger et al. 2014). A single mitochondrial genome with an average coverage of 59.83x was assembled from 7,853,206 paired-end reads using MitoFinder v. 1.4 (Allio et al. 2020), with The Invertebrate Mitochondrial Code (NCBI; transl_table = 5) specified as the organism genetic code used for translation of the 13 protein-coding genes (PCGs). Complete records for all RefSeq Decapoda Latreille, 1802 mitogenomes publicly available on NCBI were used as the reference file for MitoFinder. The assembly was annotated using the integrated MitoFinder pipeline with MEGAHIT v. 1.2.9 (Li et al. 2016) and ARWEN v. 1.2 (Laslett and Canbäck 2008) parameters, as well as the MITOS Web server (Bernt et al. 2013). Furthermore, a single circular mitochondrial genome was assembled using MITObim v. 1.9.1 (Hahn et al. 2013) and annotated using the MITOS Web server (Bernt et al. 2013); the MITObim assembly resulted in an additional 232 bp to the final contig. Both annotated assemblies were modified using Geneious 11.1.5 (http://www.geneious.com, Kearse et al. 2012). The final mitochondrial genome contains 16,212 bp and includes 13 PCGs, two rRNA genes, and 22 tRNA genes. The GC content is 26.82%. The length of the control region (CR) varies among available Axiidea mitochondrial genomes, and ranges between 546 bp (*Neaxius glyptocercus* (von Martens, 1868)) and 2036 bp (*Filhollianassa ceramica* (Fulton & Grant, 1906)). In the *E. baja* mitogenome, the largest non-coding sequence (766 bp) was located between the trn^I^ and trn^Q^ genes, which is consistent with most other members of Axiidea, and may represent the CR.

The mitochondrial gene order of *E. baja* was identical to *E. serratus* and nine of the 11 Axiidea representatives for which data were available. The mitochondrial gene order of *Trypaea australiensis* Dana, 1852 and *F. ceramica* differs from *E. baja* and other members of Axiidea in that for these two taxa, trna*^D^*, CR, and trna*^I^* are located upstream of rrnS, and the PCG nad1 is positioned after rrnL (Kou et al. 2020).

Amino acid sequences were selected for 13 mitochondrial PCGs of *E. baja* and 11 members of Axiidea, along with outgroups *Upogebia major* (De Haan, 1841 [in De Haan, 1833–1850]) and *Shinkaia crosnieri* Baba & Williams, 1998. Sequences of each PCG were aligned using MAFFT (Katoh and Standley 2013). The 13 PCG alignments were concatenated, and a maximum-likelihood tree was constructed with raxmlGUI v. 2.0.1 (Edler et al. 2021) using RaxML-NG with automatic partitions and substitution models (Figure 1). One thousand bootstrap replicate searches were used to determine node support values. These results support the monophyly of Axiidae, with the two species in *Eiconaxius* recovered as a clade that was sister to *Calocaris macandreae* Bell, 1846 [in Bell, 1844–1853].

## Data Availability

The genome sequence data that support the findings of this study are openly available in GenBank of NCBI at https://www.ncbi.nlm.nih.gov under the accession number OK448494. The associated BioProject, SRA, and Bio-Sample numbers PRJNA777625, SRX13074528, and SAMN22867214, respectively.
